# Efficacy and safety of acupuncture for urinary retention after hysterectomy: A systematic review and meta-analysis

**DOI:** 10.1097/MD.0000000000026064

**Published:** 2021-06-04

**Authors:** Qinyu Zhao, Chunchun Yan, Meng Dan, Hongling Jia

**Affiliations:** a College of Acupuncture and Tuina; b Institute of Acupuncture-moxibustion; c Institute of Chinese Medical Literature and Culture, Shandong University of Traditional Chinese Medicine; d Department of Acupuncture, The Second Affiliated Hospital of Shandong University of Traditional Chinese Medicine, Jinan, Shandong, P. R. China.

**Keywords:** acupuncture, bladder function exercise, hysterectomy, meta-analysis, systematic review, urinary retention

## Abstract

**Objective:**

The aim of this study was to evaluate the efficacy and safety of acupuncture in the treatment of urinary retention after hysterectomy in women.

**Methods:**

This research searched for 6 database documents, and the deadline is July 23, 2020. This study included a randomized controlled trial of women with urinary retention after hysterectomy. These randomized controlled trials compare acupuncture with bladder function training or other nonacupuncture treatments, and measure urodynamics, effectiveness (BR), and urinary tract infection rates (UIR). Four independent reviewers participated in data extraction and evaluation. Assess the risk of bias in each article, and conduct a meta-analysis according to the type of acupuncture. The result is expressed as a mean difference (MD) or relative risk (RR) with a 95% confidence interval (CI).

**Results:**

The meta-analysis contains 12 studies. Most studies indicate low risk or unknown risk, but the GRADE scores of the combined results show low or moderate levels. After the combined analysis, we found that acupuncture versus bladder function exercise and other nonacupuncture therapies can significantly improve the values of post voided residual urine (PVR) (MD = −25.29; 95% CI [−30.45 to −20.73]), maximal cystometric capacity (MD = 39.54; 95% CI [10.30–68.78]), bladder capacity for first voiding desire (MD = −61.98; 95% CI [−90.69 to −33.26]) and maximal flow rate (MFR) (MD = 7.58; 95% CI [5.19–9.97]). And compared with the control group, acupuncture still has advantages in BR (RR = 1.36; 95% CI [1.18–1.56]) and UIR (RR = 0.22; 95% CI [0.08–0.82]). These heterogeneities have been resolved through subgroup analysis, and their main sources are related to different intervention times, the time to start the intervention, and different PVR requirements.

**Conclusions:**

There is insufficient evidence that acupuncture can increase the patient's MFR, BR, and UIR. However, acupuncture can effectively improve the PVR, maximal cystometric capacity, and bladder capacity for first voiding desire values of patients with urinary retention after hysterectomy. Although limited due to the quality and methodological limitations of the included studies, acupuncture can still be used as an effective and safe treatment for women with urinary retention after hysterectomy.

**Registration:**

The research has been registered and approved on the PROSPERO website. The registration number is CRD42019119238.

## 1 Introduction

### 1.1 Background introduction

Hysterectomy is the removal of the uterus, sometimes involving removal of the cervix, ovaries, fallopian tubes, and other surrounding structures. In the United States, it is the second most common gynecological surgery, after cesarean section. There are many reasons for hysterectomy, such as endometriosis, adenomyosis, heavy menstrual bleeding, uterine fibroids, uterine prolapse, gynecologic cancer, transgender, and so on, but the treatment of early gynecological cancer is still its main application category. Radical hysterectomy is an effective treatment for early (I-IIa) uterine cancer.^[1]^ Cervical cancer is the fourth most common gynecological cancer in the world, and it is now found that it is mainly related to long-term infection with the HPV virus.^[1]^ Studies have calculated the incidence of cancer in 185 countries and found that in 2018 alone, there were 569,847 new cases worldwide and 311,365 cervical cancer deaths.^[2]^ China is also a country with a high incidence of cervical cancer, with 83.9% of patients undergoing hysterectomy.^[3]^ Radical hysterectomy often results in urinary retention (UR). Studies have shown that 30% to 85% of patients undergoing radical hysterectomy will have long-term bladder dysfunction (including sensory loss, incontinence, and urinary retention) after surgery.^[4,5]^ The reason for urinary retention is that when the cardinal ligament of uterus, the internal iliac vein, the lymph nodes around the deep uterine vein, the vesicouterine ligament, and the vagina are removed, the bladder autonomic nerve is cut off or injured, resulting in nerve injury bladder paralysis.^[6,7]^ The National Institute of Diabetes and Digestive and Kidney Diseases defined UR as a series of symptoms of bladder incomplete or does not empty at all. Urinary retention can be divided into acute urinary retention (AUR) and chronic urinary retention (CUR) according to the rate of onset. The European Association of Urology (EAU) and the American Urological Association (AUA) classified urinary retention in the lower urinary tract category.^[8,9]^ Nevertheless, the exact definitions of AUR and CUR remain controversial.^[10]^ Urinary retention always takes a lot of trouble such as urinary tract infection and may prolong hospital stay and increase discharge time in outpatients.^[11,12]^ Catheterization is a common way to deal with postoperative urinary retention, but catheterization also is the leading cause to increase risk of urinary tract infections, the incidence of 8%, and hospital mortality.^[13,14]^ Some reports show that Earlier removal of bladder catheter in surgical patients receiving thoracic epidural analgesia can decrease the incidence of urinary tract infection.^[15]^ Acupuncture is an effective method to decrease the incidence of urinary retention, and urinary tract infection and shortens hospital stay.^[16,17]^

### 1.2 Intervention method function introduction

Acupuncture became a hot topic in western countries about 40 years ago and has gained international fame since James Reston's piece, “Now, Let Me Tell You about My Appendectomy in Peking,” was published.^[18]^ Many scholars know that acupuncture can ease the symptoms of pain. But acupuncture is also useful in many other nonpainful diseases, which has been verified by a lot of random control trials.^[19]^ Acupuncture plays an important role in Traditional Chinese Medicine (TCM) and is commonly used for treating urinary retention in Mainland China. The AUA and GAU guidelines do not report acupuncture for urinary retention.^[8,9]^ The mechanism of the effect of acupuncture on urinary retention after nerve injury is still unclear. Its mechanism of action may involve inhibiting secondary spinal cord injury, promoting damaged spinal cord repair, regulating the neuroendocrine network of the bladder detrusor, and improving bladder function. Some studies^[20–22]^ have confirmed that acupuncture can significantly reduce the inflammatory reaction caused by urinary retention after nerve injury, inhibit the dehydration of the related neural dehydration, promote the synthesis and secretion of related neurotrophic factors, promote the repair of related nerves, restore the nerve to the bladder, and then improve the bladder function. Studies^[23]^ have also shown that electric acupuncture BL32 and BL33 can excite the afferent and efferent nerves in the rat pelvic cavity, promote detrusor contraction, and promote urination. Yi Z studies^[24]^ have shown that electroacupuncture CV4 and SP6 can reduce the myoelectric activity of the abnormal contraction of the external urethral sphincter during urination, reduce the resistance during urination, improve the coordination between the bladder detrusor and the urethral sphincter, and reduce the residual urine volume of mice with spinal cord transection. Studies^[25,26]^ conducted from the perspectives of nerves and bladder found that electroacupuncture can effectively promote the expression of nerve growth factor (NGF) and its tyrosine kinase receptor A (TrkA) in the injured spinal cord tissue of neurogenic bladder model rats. At the same time, it can significantly reduce the content of Caspase-3 in the bladder tissue. Both improve bladder function after nerve injury. Studies^[27]^ have shown that electroacupuncture may regulate the phosphorylation of factors in the detrusor muscle by activating the pituitary adenylate cyclase activating peptide (PACAP)-cAMP-PKA signaling pathway, thereby rebuilding bladder function. Studies have shown that electroacupuncture can up-regulate the expression of PACAP and protein kinase A in the rat detrusor, increase the content of cyclic adenosine monophosphate in the detrusor, promote the phosphorylation of myosin light chain kinase and myosin light chain phosphotase in the detrusor, and dephosphorylate myosin light chain, thereby promoting bladder function reconstruction. According to the meridian theory, acupuncture can cure urinary retention by regulating bladder qi transformation and promoting qi to dredge water passage. Bladder function exercise (BE) is also a method to promote the recovery of bladder function after surgery. It mainly includes training to increase abdominal pressure, basin muscle training, and intermittent catheterization. Through training, the muscles related to urination are strengthened to speed up the process of removing the catheter. Cervical cancer and other gynecological cancers have a high incidence. Radical hysterectomy, as the main method for the treatment of early gynecological cancers, often has the risk of inducing postoperative urine retention. Acupuncture is an effective method for Traditional Chinese Medicine doctors to treat urinary retention after radical hysterectomy, but there is still a lack of corresponding systematic reviews and meta-analysis reports. This study aims to assess the effectiveness and safety of acupuncture for urinary retention after hysterectomy.

## 2 Methods and materials

This systematic review and meta-analysis strictly follows the PRISMA reporting guidelines^[28]^ for design and reporting. This study is aimed at female patients with post-hysterectomy urinary retention (P), comparing acupuncture (I) and bladder training or other non-acupuncture treatments (C), observing the results of urodynamics (O), and evaluating the improvement and safety of acupuncture treatment of post-hysterectomy urinary retention. The research has been registered and approved on the PROSPERO website (CRD42019119238), and the corresponding protocol has been published in *Medicine*.^[29]^ Each step of this review strictly refers to the Cochrane Handbook 5.2.

### 2.1 Study selection

Search for published literature in databases including PubMed, Web of Science, Cochrane Controlled Trial Center Registration, China National Knowledge Infrastructure (CNKI), Wanfang Data, Chongqing VIP Database, and the language of the literature is not limited. From the establishment of the database to July 23, 2020, a literature search was conducted on the available publications to find appropriate randomized controlled trials of acupuncture for the treatment of urinary retention after hysterectomy. The search strategies used a combination of the terms “acupuncture,” “electroacupuncture,” and “urinary retention,” We provide a more detailed search strategy in Supplementary Material (see Appendix 1, Supplemental Content, which provides a more detailed search strategy).

### 2.2 Inclusion/exclusion criteria

We studied the effect of acupuncture on urodynamic indexes of patients with urinary retention after hysterectomy. The control group included BE, sham acupuncture (SA) with BE, or oral brompistigmine (B) with BE. The literature that acupuncture and bladder exercise are not the main treatment methods and other related research literature are not included. This meta-analysis included only published clinical randomized controlled trials. The detailed literature selection criteria are in the attachment (see Appendix 2, Supplemental Content, which provides detailed literature selection criteria). Two independent reviewers (QYZ and CCY) screened the research results according to the eligibility criteria, conducted a preliminary screening based on the title and abstract, and removed duplicate documents. Any disagreement will be discussed and resolved by the third reviewer (HLJ). All the selected studies were further reviewed.

#### 2.2.1 Outcomes of interests

The urodynamic examination can reflect the function of the bladder, and thus reflect the bladder recovery of patients with urinary retention after hysterectomy. Therefore, we selected post voided residual urine (PVR), maximal cystometric capacity (MCC), maximal flow rate (MFR), and bladder capacity for first voiding desire (BFD) in urodynamic indicators as the primary outcomes. The secondary outcomes included bladder function recovery rate (BR) and urinary tract infection rate (UIR). Safety outcomes are reports of related adverse events.

### 2.3 Data extraction

The data are extracted from the full-text article through a pre-designed literature feature table, which includes participant features, intervention measures, comparisons, checkpoints, and main results. Two reviewers (QYZ and CCY) independently evaluate the eligibility and quality of the research. If they have different opinions, they will decide after discussion and consensus.

### 2.4 Assessment of risk of bias

All studies were evaluated for risk of bias in strict accordance with the Cochrane Handbook for Systematic Reviews of Interventions.^[30]^ The assessment of the risk of bias in each study was carried out simultaneously and separately by 2 reviewers (QYZ and CCY). If 2 reviewers disagree on the risk of bias in research, they will reach a consensus through consultation.

### 2.5 Statistical analysis

Quantitative analysis was performed by meta-analysis using Cochrane collaboration software RevMan 5.3.5. The relative risk (RR) was selected as the statistic for dichotomous data; the continuous variables are described using mean difference (MD) and 95% confidence interval (CI). According to the number of interventions, checkpoints and different efficiency standards, a subgroup analysis was carried out. The MFR levels showed as mL/s, and PVR, MCC, and BFD showed as milliliter. We evaluated the heterogeneity between studies by using Cochran *Q* statistic with the associated *P* value. Quantify the degree of heterogeneity by measuring *I*^*2*^. A fixed-effect model was used when *I*^*2*^ was ≤50% and the *P* value was ≥.10; when *I*^*2*^ was >50% or the *P* value was <.10, random-effect model was applied. *I*^*2*^ < 50% and *P* > .10 indicated that all of the studies were homogeneous; *I*^*2*^ > 75% suggested the heterogeneity was high; *I*^*2*^ between 50% and 75% suggested the heterogeneity was moderate.^[31]^ We explore the source of heterogeneity through subgroup analysis or sensitivity analysis. The statistical significance was set as *P* < .05.

### 2.6 Quality of evidence

The level of evidence quality of all results was assessed according to GRADE (Grading of Recommendations Assessment, Development, and Evaluation).^[32]^

## 3 Results

### 3.1 Study selection

**Figure 1:**
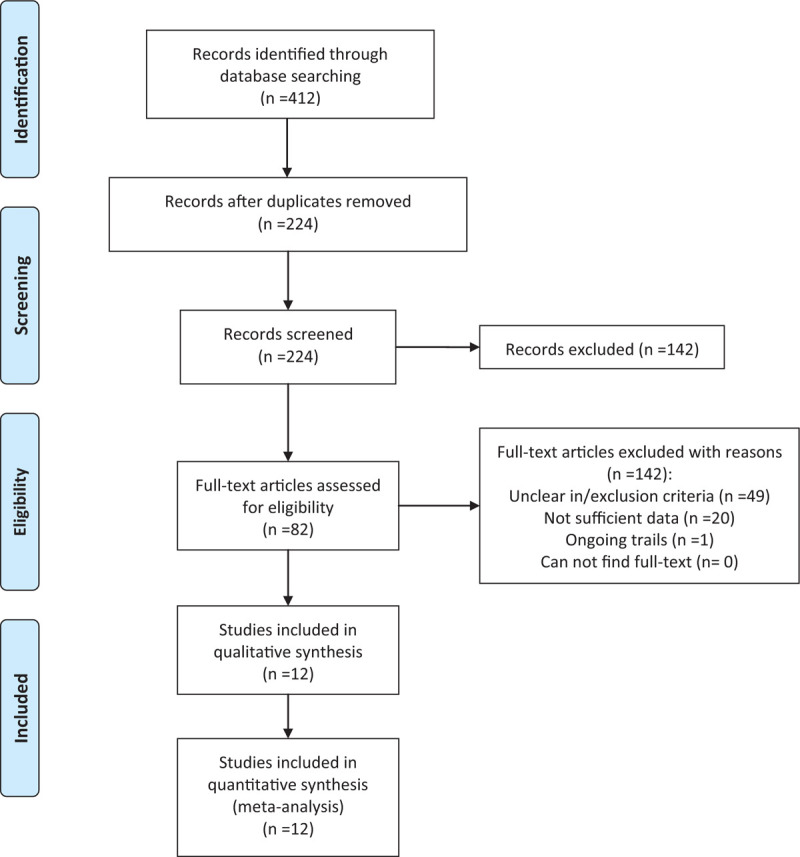
PRISMA flow chart of literature retrieval and study selection.

The PRISMA flow chart of study selection is shown in Figure 1. A total of 412 records were retrieved in the first search, and 224 records were retained after removing duplicates. After screening the title or abstract, 142 records were removed. The remaining 82 articles were selected as full-text reviews. Finally, 12 studies were included in this systematic review and meta-analysis.

### 3.2 Study characteristics

The characteristics of all included studies are in Table 1. In these 12 studies, all trails were conducted in China.^[33–44]^ Of the 12 trails, one^[39]^ was published in English and the rest^[33–38,40–44]^ were published in Chinese. One^[41]^ is a doctorate thesis, and the remaining 11^[33–40,42–44]^ are peer-reviewed articles. One^[37]^ is a multicenter study, but the sample size is not very large. The sample size ranged from 58 to 192, with a total of 1271 participants. The duration of therapy varied from 5 days to 1 month. All studies compared the baseline data of each group of patients, and there was no difference (*P* > .05).

**Table 1:** Characteristic of included studies.

	Sample size		Age						
Author, year	Acup	Ctrl	Acup	Ctrl	Acup type	comparison	Acup-session	Checkpoint	main outocmes
^∗^Yi et al, 2008^[33]^	55	55	47.5 ± 7.8	46.3 ± 8.2	EA + BE	BE	1/day for 5 days, 8 days after surgery	19 Days after surgery	PVR, MCC
Qiu et al, 2010^[34]^	31	31	N/A	N/A	EA + BE	BE	1/day for 5 days, 7 days after surgery	14 Days after surgery	PVR, BR
^†^Jiang et al, 2010^[35]^	64	64/64	N/A	N/A	LIA + BE	BE	1/day for 6 days, 8 days after surgery	15 Days after surgery	PVR, BR, AE
^‡^Hu et al, 2012^[36]^	19/20	19	43.63 ± 8.10	41.95 ± 5.38	EA + BE	BE	1/day for 6 days, 8 days after surgery	14 Days after surgery	PVR, BR
^§^Zeng et al, 2014^[37]^	60	62/58	58.7 ± 14.2	57.3 ± 11.5	EA + BE	BE	1/day for 7days, 5 days after surgery	14 Days after surgery	MCC, MFR, BR, BFD
Ye and Zhao, 2014^[38]^	74	33	46.92 ± 13.69	46.26 ± 12.89	EA + BE	BE	1/day for 10 days, 4 days after surgery	14 Days after surgery	PVR, BR, AE
^¶^Yi et al, 2014^[39]^	60	60	46.5 ± 7.7	45.9 ± 8.2	EA + RC	SA + RC	1/day for 5 days, 6 days after surgery	15/30 Days after surgery	PVR, MCC, MFR, AE
Zhao et al, 2015^[40]^	30	30	63.7 ± 3.2	64.2 ± 2.7	EA + BE+P	BE+P	1/day for 20 days, 14 days after surgery	34 Days after surgery	PVR, MCC, MFR, BR
^||^Ding, 2017^[41]^	30/30/30/30	30	47.27 ± 8.05/47.73 ± 8.09/49.70 ± 9.24/47.43 ± 10.13	48.70 ± 9.27	BE + (Acup/Acup + SCA)	BE	1/day for 10 days, 2 days before or after surgery	1/3/6 mo after surgery	PVR, MFR, BFD, AE
Liu and Li, 2018^[42]^	48	48	47.36 ± 6.91	46.85 ± 7.12	EA + AI (P) + RC	RC	1/day	14 Days after surgery	PVR, BR
Yang, 2019^[43]^	36	36	45.11 ± 13.68	46.10 ± 12.88	EA + BE	BE	1/day for 5 days, 5 days after surgery	Remove the catheter for 4–6 h	PVR, BR, UIR
Peng et al, 2019^[44]^	32	32	49.9 ± 19.9	48.9 ± 19.7	EA +BE	BE	1/day for 1 mo, 5 days after surgery	35 Days after surgery	MFR, BR, UIR

### 3.3 Judgment on the risk of bias

All studies were critically evaluated by 2 independent reviewers for random sequence generation, allocation concealment, blinding of participants and personnel, blinding of outcome assessment, incomplete outcome data, selective reporting, and other sources of bias. We divide each part into high risk, unclear risk, and low risk. The assessment of the risk of bias mainly has the following criteria: high risk (although there is a corresponding description, it fails to meet the standard in the description of the operation process); unclear risk (no specific circumstances or relevant information mentioned); low risk (meet the corresponding standards and have corresponding specific instructions)

#### 3.3.1 Random sequence generation

Six trails^[33,35,36,38,39,42]^ were rated as low-risk, 4^[33,35,36,42]^ of which described using the random number table method, and 2^[38,39]^ described using a computer-generated random number method. One^[44]^ was rated as high-risk because the study description would include patients for equalization. The remaining 5 studies^[34,37,40,41,43]^ were rated as an unclear risk because they only described random, but did not describe the specific methods of random in detail.

#### 3.3.2 Allocation concealment

There is only 1 study^[39]^ on the risk of bias of allocation concealment as low risk, which details the use of opaque envelopes for allocation concealment. Three studies^[33,35,41]^ were rated as high-risk because they all mentioned grouping in the order of surgery. The remaining 9 studies^[34,36–38,40,42–44]^ were rated as low risk because they did not specifically describe the method of allocation concealment.

#### 3.3.3 Blinding of participants and personnel

Since acupuncture requires the doctor to operate it personally and the pain caused by acupuncture needles piercing the skin is often difficult to implement blinding, the score is generally low. Only 1^[39]^ was rated as low risk, which specifically described the implementation of blinding by separating patients from different groups and using sham acupuncture. The remaining 11 trails^[25–38,40–44]^ did not describe whether to use separate treatment of different groups of patients or other blinding methods and were rated as an unclear risk.

#### 3.3.4 Blinding of outcomes assessment

A study^[39]^ described sending the data to a third party for analysis and confirmed in advance that the specific analysis method was rated as low risk. A study^[44]^ was rated as high risk because it did not specifically describe the analysis methods and standards of the corresponding data. The remaining 11 studies^[25–38,40–43]^ described specific analysis methods and standards but did not describe whether blind methods such as third-party analysis were used, and were rated as unclear risks.

#### 3.3.5 Incomplete outcome data

There are 2 trails^[39,41]^ describing the drop-outs and describing the detailed reasons for drop-outs, which are defined as low risk. The remaining 10 trails^[25–38,40,42–44]^ were not reported to drop-outs and were defined as unclear risks.

#### 3.3.6 Selective reporting

Six trails^[33,35,36,38,39,41]^ were rated a low risk of bias with all the excepted outcomes reported. Other 6 trials^[34,37,40,42–44]^ were at unclear risk of bias because they reported all the outcomes but without adverse effect.

#### 3.3.7 Other bias

A study^[41]^ is defined as high risk because it is the risk of possible publication and result bias in the doctoral dissertation. Five studies^[34,36,39,42,44]^ were rated as low risk because they all have specific funds to support the research. The remaining studies^[33,35,37,38,40,43]^ are defined as unclear risks because they have no fund support.

### 3.4 Validity of included studies

The risk of bias for all included studies is shown in Figure S1 (see Figure S1, Supplemental Content, which provides the risk of bias graph and summary). The risk of bias in most studies is either unclear or high. This was mainly because blinding was not carried out or mentioned in the randomization and allocation process, and it was difficult to use blinding during acupuncture intervention. The summary of the evidence credibility of the corresponding results is provided in the GRADE Certainty of Evidence Profiles (see Table S1, Supplemental Content, which provides detailed GRADE scores).

### 3.5 Outcomes

We report the results separately according to the outcome type.

### 3.6 PVR

**Figure 2:**
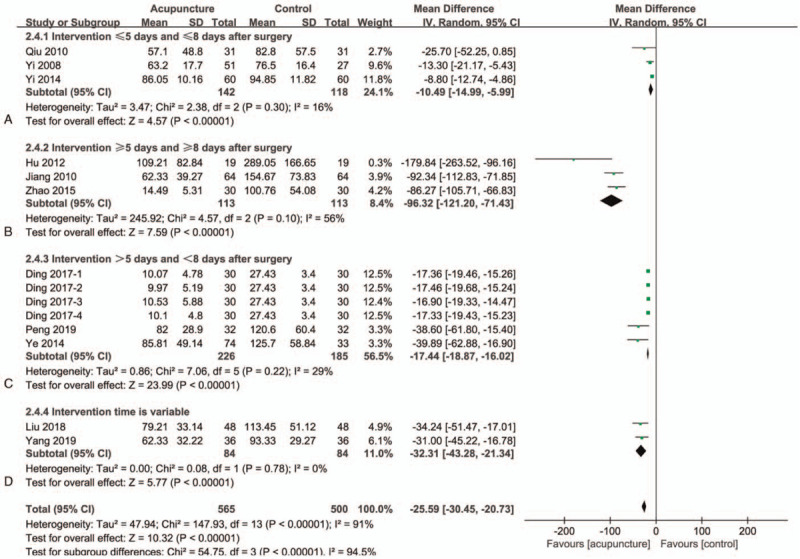
Forest plots of meta-analysis outcomes of post voided residual urine (PVR). CI = confidence interval. Test for heterogeneity, Chi-Squared statistic with its degrees of freedom (d.f.) and *P* value; *I*^*2*^, inconsistency among results; test for overall effect, Z statistic with P value.

There are 11 studies^[33–36,38–44]^ in this group with a total of 975 participants (Fig. 2). In the included studies, the combined MFR values showed significant differences between the 2 groups, but with high heterogeneity (MD = −25.29; 95% CI −30.45 to −20.73]; *I*^*2*^ = 91%; *P* < .00001) (GRADE certainty of evidence: low). Through our analysis, it is found that the length of the acupuncture intervention and the time to start the intervention have an impact on the heterogeneity, and the subgroup analysis based on these has effectively resolved the heterogeneity. We included the 3 studies^[33,34,39]^ that started intervention within 8 days after the operation and the intervention was ≤5 days into part A (Fig. 2A) section. These studies have significant differences between the 2 groups and are homogeneous (MD = −10.49; 95% CI −14.99 to −5.99]; *I*^*2*^ = 16%; *P* < .00001). The other 3 studies^[35,36,40]^ that started 8 days after the operation and the intervention was ≥5 days included part B (Fig. 2B). These studies still have significant differences between the 2 groups, and have moderate heterogeneity (MD = −96.32; 95% CI −121.2 to −71.43]; *I*^*2*^ = 56%; *P* < .00001). There are 3 other studies, which started within 8 days after surgery and the intervention time is >5 days, and we included them in part C (Fig. 2C). These studies also have significant differences between the 2 groups and are homogeneous (MD = −17.44; 95% CI −18.87 to −16.02]; *I*^*2*^ = 29%; *P* < .00001). One study^[42]^ did not specify the length of treatment, and the other study had a different treatment end time for each patient. These 2 studies were included in part D (Fig. 2D). After the 2 studies were combined, there were significant differences and homogeneity (MD = −32.31; 95% CI −43.28 to −21.34]; *I*^*2*^ = 0%; *P* < .00001). It can be seen that the source of heterogeneity of PVR comes from when the intervention and the length of time of acupuncture intervention, and it can be clearly seen from the CI value that the earlier the intervention, the longer the intervention time, the more beneficial the improvement of the PVR value.

### 3.7 MCC

**Figure 3:**
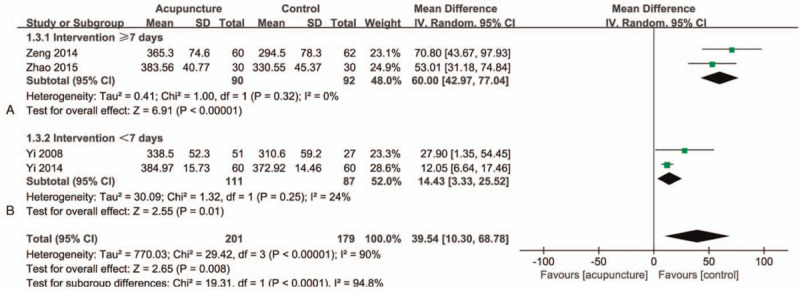
Forest plots of meta-analysis outcomes of maximal cystometric capacity (MCC). CI = confidence interval. Test for heterogeneity, *χ*^2^ statistic with its degrees of freedom (d.f.) and *P* value; *I*^*2*^, inconsistency among results; test for overall effect, *Z* statistic with *P* value.

There are 4 studies^[33,37,39,40]^ in this group with a total of 380 participants (Fig. 3). We combined the 4 studies and found that MCC has differences between groups, but the heterogeneity is large (MD = 39.54; 95% CI 10.30–68.78]; *I*^*2*^ = 90%; *P* = .008) (GRADE certainty of evidence: low). It is found through observation that the length of the intervention period may be the source of heterogeneity. We set the intervention time as a boundary of 7 days and divided the 4 studies into 2 groups A and B, which effectively resolved the heterogeneity. The intervention time of the 2 studies^[37,40]^ is ≥7 days, and there are significant differences and homogeneity after the merger (MD = 60.00; 95% CI 42.97–77.04]; *I*^*2*^ = 0%; *P* < .00001) (Fig. 3A). The remaining 2 studies^[33,39]^ have an intervention time of <7 days, and there were still group differences and homogeneity after the merger (MD = 14.43; 95% CI 3.33–25.52]; *I*^*2*^ = 0%; *P* = .01) (Fig. 3B). We can easily see that for MCC, its heterogeneity comes from the difference in the intervention time. By observing the CI value, it can be seen that the longer the intervention time, the more beneficial the improvement of the MCC value.

### 3.8 MFR

**Figure 4:**
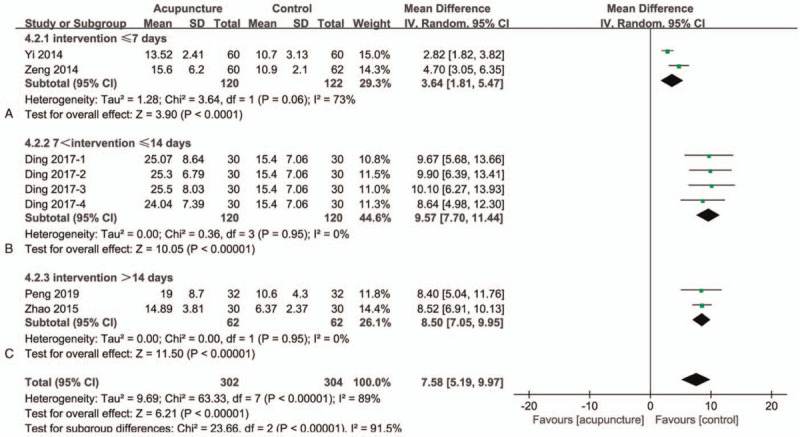
Forest plots of meta-analysis outcomes of maximal flow rate (MFR). CI = confidence interval. Test for heterogeneity, *χ*^2^ statistic with its degrees of freedom (d.f.) and *P* value; *I*^*2*^, inconsistency among results; test for overall effect, *Z* statistic with *P* value.

There are 5 studies^[37,39–41,44]^ in this group with a total of 516 participants (Fig. 4). One of which had 4 acupuncture arms with different points and intervention time. We compared 4 arms with control group, respectively. The overall heterogeneity of all the studies combined is large, but there are still significant differences between the 2 groups (MD = 7.58; 95% CI 5.19–9.97]; *I*^*2*^ = 89%; *P* < .00001) (GRADE certainty of evidence: very low). We divided the 5 included studies into groups according to the intervention time and checkpoint of the acupuncture group and performed subgroup analysis. The 2 studies^[37,39]^ were divided into part A, and their intervention time was <7 days (Fig. 4A). After the 2 studies are combined, the heterogeneity is moderate, and the two groups have significant differences (MD = 3.64; 95% CI 1.81–5.47]; *I*^*2*^ = 73%; *P* < .0001). Only Ding's acupuncture intervention time was between 7 and 14 days, which was classified as Part B (Fig. 4B), and the 2 groups showed significant differences and homogeneity (MD = 9.57; 95% CI 7.70–11.44]; *P* < .00001). The remaining 2 studies^[40,44]^ were divided into part C (Fig. 4C) with an intervention time >14 days. There are still big differences between the 2 groups, and they are homogeneous after the merger (MD = 8.50; 95% CI 7.05–9.95]; *I*^*2*^ = 0%; *P* < .00001). The huge heterogeneity after the merger may be related to the inconsistency of the intervention time between the studies, and the improvement of MFR is proportional to the intervention time.

### 3.9 BFD

**Figure 5:**
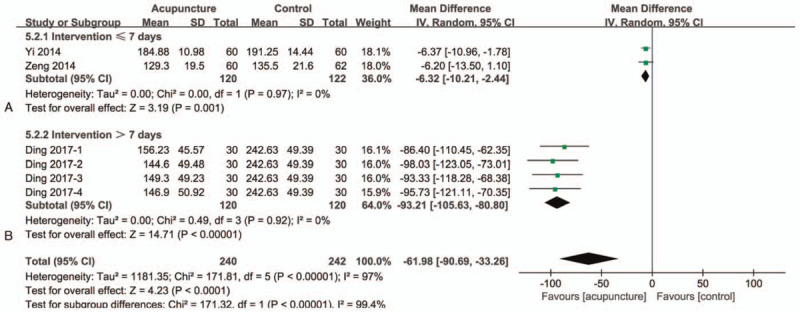
Forest plots of meta-analysis outcomes of bladder capacity for first voiding desire (BFD). CI = confidence interval. Test for heterogeneity, *χ*^2^ statistic with its degrees of freedom (d.f.) and *P* value; *I*^*2*^, inconsistency among results; test for overall effect, *Z* statistic with *P* value.

There are 3 studies^[37,39,41]^ in this group with a total of 392 participants (Fig. 5). One of which had 4 acupuncture arms with different points and intervention time. We compared 4 arms with control group respectively. After the 3 studies were combined, the BFD values showed significant differences between the 2 groups, and the overall heterogeneity was high (MD = −61.98; 95% CI −90.69 to −33.26]; *I*^*2*^ = 97%; *P* < .00001) (GRADE certainty of evidence: low). To solve the heterogeneity, we divide the intervention time into 2 parts A and B based on the intervention time of 7 days. Part A (Fig. 5A) is ≤7 days, there are 2 studies,^[37,39]^ and Part B (Fig. 5B) is >7 days, there is one study.^[41]^ There are significant differences between the 2 groups after the merger of Part A, and they are homogeneous (MD = −6.32; 95% CI −10.21 to −2.44]; *I*^*2*^ = 0%; *P* = .001). Part B is the same as Part A after merging, with significant differences between groups and homogeneity (MD = −93.21; 95% CI −105.63 to −80.80]; *I*^*2*^ = 0%; *P < *.00001). Therefore, we can easily see that the heterogeneity of this part also comes from the length of the intervention time, and the improvement of BFD is also positively correlated with the length of the intervention.

### 3.10 BR

**Figure 6:**
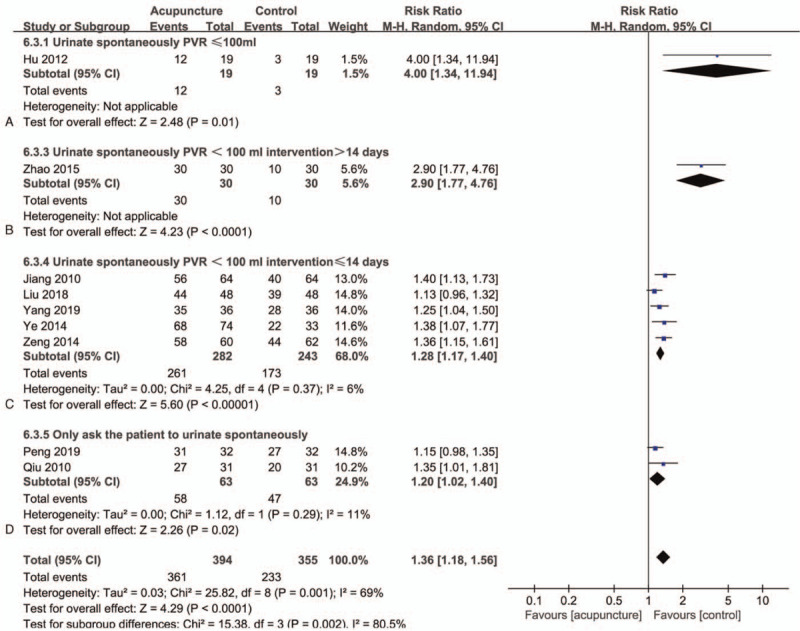
Forest plots of meta-analysis outcomes of bladder function recovery rate (BR). CI = confidence interval. Test for heterogeneity, *χ*^2^ statistic with its degrees of freedom (d.f.) and *P* value; *I*^*2*^, inconsistency among results; test for overall effect, *Z* statistic with *P* value.

There are 9 studies^[34–38,40,42–44]^ in this group with a total of 749 participants (Fig. 6). After combining all the studies, there are significant differences between the 2 groups, but the overall heterogeneity is moderate (RR = 1.36; 95% CI 1.18–1.56]; *I*^*2*^ = 69%; *P < *.0001) (GRADE Certainty of evidence: very low). Regarding the definition of BR, all studies require patients to urinate autonomously to be judged as effective. However, some studies have other requirements on PVR, so preliminary grouping is required according to the different requirements for PVR, and then grouping again according to the intervention time. We divided the included studies into four groups based on PVR and intervention time. Only Hu et al^[36]^ is classified in part A (Fig. 6A), and there are differences between the 2 groups (RR = 4.00; 95% CI 1.34–11.94]; *P* = .01). Zhao et al^[40]^ was placed in part B (Fig. 6B), and the comparison also showed significant differences (RR = 2.90; 95% CI 1.77,4.76]; *P* < .0001). Seven studies^[35,37,38,42,43]^ were divided into part C (Fig. 6C), there are significant differences between the groups, and the heterogeneity after merging is small (RR = 1.28; 95% CI 1.17–1.40]; *I*^*2*^ = 6%; *P* < .00001;). The remaining 2 studies^[34,44]^ are divided into part D (Fig. 6D). These 2 studies only required patients to urinate spontaneously, without further requirements for PVR. There are differences between the groups, and the combined heterogeneity is also small. (RR = 1.20; 95% CI 1.02–1.40]; *I*^*2*^ = 11%; *P* = .02). From this point of view, the heterogeneity of BR is not only derived from the subtle differences in PVR requirements but also affected by the intervention time.

### 3.11 UIR

**Figure 7:**
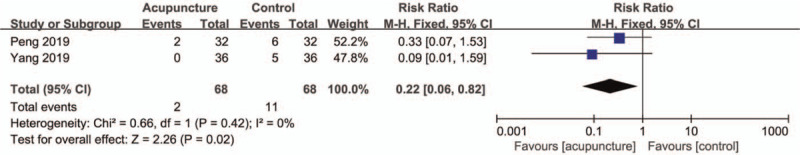
Forest plots of meta-analysis outcomes of urinary tract infection rate (UIR). CI = confidence interval. Test for heterogeneity, *χ*^2^ statistic with its degrees of freedom (d.f.) and *P* value; *I*^*2*^, inconsistency among results; test for overall effect, *Z* statistic with *P* value.

Two studies^[43,44]^ are included in this part of the study with 136 participants, there are significant differences between the groups and small heterogeneity after merging (Fig. 7). Regarding the determination of UIC, the 2 studies did not clearly propose the criteria but simply said whether there was urinary tract infection. Besides, only 2 articles were included, so a cautious attitude should be adopted for this result. After the UIR data are merged, it is shown that there are significant differences between the groups, and the merged data have good homogeneity (RR = 0.22; 95% CI 0.08–0.82]; *I*^*2*^ = 0%; *P* = .02) (GRADE certainty of evidence: low).

## 4 Discussion

### 4.1 Summary of main findings

This article aims to summarize and evaluate the effects of acupuncture treatment on changes in PVR, MCC, MFR, BFD, BR, and UIR in patients with urinary retention after hysterectomy. In general, we found that acupuncture combined with BE compared with BE or other nonacupuncture treatments has significant differences in PVR, MCC, MFR, BFD, BR, and UIR in patients with urinary retention after hysterectomy (*P* < .05). The corresponding research results of UIR have good homogeneity (*I*^*2*^ = 0%) after the merger, but there is moderate heterogeneity (*I*^*2*^ = 69%) after the corresponding study of BR, and the corresponding research of PVR, MCC, MFR, BFD has higher heterogeneity (*I*^*2*^ > 89%) after the merger. I mainly performed a subgroup analysis based on the course of treatment to solve the heterogeneity of MCC, MFR, and BFD, and performed a subgroup analysis based on the course of treatment and the start of intervention time to solve the heterogeneity of PVR. We have rigorously reviewed the criteria for BR and UIR. Their criteria are roughly the same and are also in line with the clinical situation. According to the PVR value for further requirements for efficiency, a subgroup analysis of BR is performed to resolve heterogeneity. As the GRADE scores for the level of evidence of each indicator are between “Low” and “Very Low,” we should be more cautious about the results of PVR, MCC, MFR, BFD, BR, and UIR. Through sensitivity analysis, it is found that the heterogeneity of BR value is affected by a single study,^[40]^ and the heterogeneity is reduced from moderate (*I*^*2*^ = 69%) to low heterogeneity (*I*^*2*^ = 40%). The reason should be related to the late acupuncture intervention in this study^[40]^ (within 5 days after surgery *vs.* 14 days after surgery). MCC and MFR were also affected by the same study^[39]^ in sensitivity analysis, and their heterogeneity decreased from high (*I*^*2*^ = 90%) to moderate (*I*^*2*^ = 60%). Through analysis, we found that this study is different from other studies in that it observes the long-term effect of acupuncture on patients (there is a 19-day interval between the end of treatment and the examination time). Sensitivity analysis showed that any other combined results were not excessively affected by any single trial, and any study did not affect the difference between the 2 groups (see Table S2, Supplemental Content, which provide detailed sensitivity analysis results). Combined with our discussion of the subgroup analysis and classification methods of various indicators in the “outcomes” section of this article, it is not difficult to see that these indicators are related to the length of the intervention period and whether the intervention is carried out in the early stage. Through sensitivity analysis, it can also be found that acupuncture intervention has a certain long-term effect. We also found that the earlier the acupuncture intervention time, the longer the acupuncture intervention duration, the more beneficial it is for the improvement of various indicators. This is the same as other therapies for the treatment of other diseases and requires early intervention and sufficient amounts.

### 4.2 Applicability of current evidence

Although some results have good homogeneity after merging, we still need to treat these results with caution. Although the inclusion of patients with urinary retention stated that patients could not urinate spontaneously and indwell a catheter, there are some differences in some subtle regulations, such as one study^[40]^ with a postoperative 14 days ≥100 mL requirements. When calculating the effective rate, although all require patients to urinate autonomously, some details are slightly different. Most require that the residual urine volume must be <100 mL, but a few^[34,44]^ require autonomous urination even if the residual urine volume >100 mL is effective. The number of interventions of acupuncture and the time to start the intervention is also different. Most of the research interventions are about 7 times, most of them start the intervention about 1 week after the operation, but a few study^[40,44]^ interventions can reach up to 30 times, and the intervention may start 2 weeks after the operation. The checkpoint for various tests is also different. Most of them are checked 2 weeks after surgery, and a small part^[40,41,44]^ is checked 1 month or even 6 months after surgery. Other factors such as acupuncture points and acupuncture expertise also affect the results. Although we conducted subgroup analysis and sensitivity analysis based on the above issues to eliminate heterogeneity as much as possible and make the data evidence more reliable, we still need to treat these combined results with caution.

### 4.3 Strengths and limitations of this study

Although there are some systematic reviews and meta-analysis reports on acupuncture treatment of urinary retention, specific reports on patients with urinary retention caused by hysterectomy have not been found. Urinary retention caused by hysterectomy is significantly different from other causes in the pathogenesis of urinary retention. Therefore, further research on urinary retention after hysterectomy is needed. There are 2^[45,46]^ systematic reviews and meta-analysis on the treatment of neurogenic bladder with acupuncture after spinal cord injury. The sex of the patients includes male, and the nature of the injury is urinary retention caused by central nerve injury. These studies are different from ours in terms of patient type, pathogenesis, length of disease, and difficulty of recovery. A study^[47]^ reported on the role of acupuncture in promoting recovery after gynecological surgery. The focus is on all the symptoms that promote postoperative recovery, and no special attention has been paid to postoperative urinary retention. Two studies^[48,49]^ reported a systematic review and meta-analysis of acupuncture interventions in the treatment of postpartum urinary retention. The causes of postpartum urinary retention are mostly related to the suppression of urination reflex caused by anesthetics, and reflex spasm of the bladder and posterior urethral sphincter caused by wound pain. Because the uterus and surrounding tissues are not removed after delivery, it is unlikely to damage the bladder autonomic nerve and cause urinary retention. Therefore, postpartum urinary retention is different from our research topics in many aspects, such as pathogenesis, recovery time, and recovery difficulty. So we conducted this study, but considering that some low-quality studies may be biased, we also conducted a sensitivity analysis and got the corresponding results. However, due to limitations, caution should be exercised when interpreting these results.

### 4.4 Implications for practice and research

Considering the benefits of acupuncture demonstrated in this study, acupuncture may be a therapy for the treatment of urinary retention after hysterectomy. Preoperative intervention for patients who are about to undergo hysterectomy can also effectively shorten the time of postoperative urine retention. After the merger and sensitivity analysis, there are differences between acupuncture and the control group, but there are different degrees of heterogeneity. The length of treatment and whether to conduct early intervention is the source of the heterogeneity of various indicators. Subgroup analysis for them can effectively resolve the heterogeneity. Therefore, follow-up research on acupuncture treatment of urinary retention can focus on the length of intervention and whether to conduct the early intervention. The GRADE scores for these indicators are “Low” or “Very Low.” Regarding the risk of bias, acupuncture-related research needs further design in terms of “Allocation concealment” and “Blinding of outcomes assessment.” Nevertheless, larger and better-designed clinical trials are still needed to clarify the effect of acupuncture on UR after hysterectomy.

## Author contributions

The manuscript is written by Q.Y. Zhao; Q.Y. Zhao conceived the systematic review; Q.Y. Zhao and C.C. Yan performed literature searches, study selection, data extraction, risk of bias assessment, meta-analysis and wrote the initial draft; Q.Y. Zhao and D. Meng conducted statistical analysis; all of the authors critically revised the manuscript.

**Conceptualization:** Hongling Jia, Meng Dan.

**Data curation:** Chunchun Yan.

**Funding acquisition:** Hongling Jia.

**Investigation:** Hongling Jia, Chunchun Yan.

**Methodology:** Qinyu Zhao, Hongling Jia, Meng Dan.

**Software:** Meng Dan.

**Writing – original draft:** Qinyu Zhao, Chunchun Yan.

**Writing – review & editing:** Qinyu Zhao, Chunchun Yan.

## Supplementary Material

Supplemental Digital Content

## Supplementary Material

Supplemental Digital Content

## Supplementary Material

Supplemental Digital Content

## Supplementary Material

Supplemental Digital Content

## Supplementary Material

Supplemental Digital Content
