# Supplementary material for: Efficacy and safety of acupuncture for urinary retention after hysterectomy: A systematic review and meta-analysis
Source: Medicine (Baltimore). 2021 Jun 4;100(22):e26064. doi: 10.1097/MD.0000000000026064 (PMC8183752; doi:10.1097/MD.0000000000026064)
Supplement: Supplemental Digital Content [file medi-100-e26064-s001.doc]

**Appendix 1. Search strategy in Pubmed**

#1 Hysterectomy [Title/Abstract]

#2 Hysterectomies [Title/Abstract]

#3 #1 OR #2

#4 Uterine Hemorrhage [Title/Abstract]

#5 Uterine Hemorrhages [Title/Abstract]

#7 Hemorrhage, Uterine [Title/Abstract]

#9 Uterine Bleeding [Title/Abstract]

#10 Bleeding, Uterine [Title/Abstract]

#11 Uterine Bleedings [Title/Abstract]

#12 Vaginal Bleeding [Title/Abstract]

#13 Bleeding, Vaginal [Title/Abstract]

#14 Bleedings, Vaginal [Title/Abstract]

#15 Vaginal Bleedings [Title/Abstract]

#16 #4 OR #5 OR #6 OR #7 OR #9 OR #10 OR #11 OR #12 OR #13 OR #14 OR #15

#17 endometriosis [Title/Abstract]

#18 uterine fibroids [Title/Abstract]

#19 Heavy menstrual bleeding [Title/Abstract]

#20 Uterine prolapse [Title/Abstract]

#21 Reproductive system cancer prevention [Title/Abstract]

#22 Gynecologic cancer [Title/Abstract]

#23 Transgender [Title/Abstract]

#24 Severe developmental disabilities [Title/Abstract]

#25 Chronic pelvic pain [Title/Abstract]

#26 #17 OR #18 OR #19 OR #20 OR #21 OR #22 OR #23 OR #24 OR #25

#27 #16 OR #26

#28 acupuncture therapy [Title/Abstract])

#29 acupuncture-moxibustion [Title/Abstract]

#30 meridian*[Title/Abstract]

#31 electroacupuncture [Title/Abstract]

#32 #28 OR #29 OR #30 OR #31

#33 acupoint [Title/Abstract]

#34 acupuncture Points [Title/Abstract]

#35 acupressure [Title/Abstract]

#36 acupressure-acupuncture therapy [Title/Abstract]

#37 #33 OR #34 OR #35 OR #36

#38 warm needling [Title/Abstract]

#39 moxa needle [Title/Abstract]

#40 acupuncture plus moxibustion [Title/Abstract]

#41 moxibustion with warming needle [Title/Abstract]

#42 #38 OR #39 OR #40 OR #41

#43 auricular acupuncture [Title/Abstract]

#44 auricular needle [Title/Abstract]

#45 ear acupuncture [Title/Abstract]

#46 moxibustion [Title/Abstract]

#47 #43 OR #44 OR #45 OR #46

#48 abdom* acupuncture [Title/Abstract]

#49 #32 OR #37 OR #42 OR #47 OR #48

#50 urinary retention [Title/Abstract]

#51 retention, Urinary [Title/Abstract]

#52 #51 OR #52

#53 (#3 OR #27) AND #49 AND #51

#54 Hysterectomy [MeSH Terms] OR Uterine Hemorrhage [MeSH Terms]

#55 Acupuncture [MeSH Terms]

#56 urinary retention [MeSH Terms]

#57 #54 AND #55 AND #56

#58 #53 OR #57
