# Supplementary material for: Efficacy and safety of acupuncture for urinary retention after hysterectomy: A systematic review and meta-analysis
Source: Medicine (Baltimore). 2021 Jun 4;100(22):e26064. doi: 10.1097/MD.0000000000026064 (PMC8183752; doi:10.1097/MD.0000000000026064)
Supplement: Supplemental Digital Content [file medi-100-e26064-s002.doc]

**Appendix 2. Study selection**

This study is aimed at female patients with post-hysterectomy urinary retention (P), comparing acupuncture (I) and bladder training (C), observing the results of urodynamics (O), and evaluating the improvement and safety of acupuncture treatment of post-hysterectomy urinary retention. We compared acupuncture with bladder function exercise, bladder function exercise with fake acupuncture and bladder function exercise with oral Pyrazine bromide.

Only published RCTs were included in this meta-analysis. Literatures in which review, non-clinical controlled experiments, animal experiments, case reports, clinical experience reports, acupuncture and bladder function exercise were not the main research objects, incorrect literature or data were excluded. Other therapies such as simple moxibustion, acupoint pressing, and thread embedding were also excluded. Literatures that did not clearly include and exclude criteria, did not publish enough data, only published abstracts but could not obtain the full text, and have not yet finished clinical trials, will be excluded in a later step.

***Type of studies***

We chose to include clinical randomized controlled trials (RCTs) evaluating the efficacy of acupuncture in the treatment of post-hysterectomy urinary retention. Non-clinical controlled experiments, animal experiments, case reports, clinical experience reports, acupuncture and bladder function exercise were not the main research objects were excluded.

***Type of participants***

The inclusion criteria for participants included: 1) Patients undergoing hysterectomy. 2) Patients diagnosed with urinary retention after hysterectomy. 3) Patients who receive acupuncture and bladder function exercise as the main treatment.

***Type of interventions***

We included the trials in which acupuncture needles (with or without electrical stimulation) were inserted into or laser Irradiated traditional acupoints. Trials using acupuncture-related techniques which did not include skin penetration, such as moxibustion, acupressure, auricular point sticking, catgut embedding were excluded. The literature that acupuncture is not the main treatment research object is not included in this study.

***Type of control group***

We included bladder function exercise, bladder function exercise combined with fake acupuncture and bladder function exercise combined with oral Pyrazine bromide as the control group.

***Type of outcome measures***

We select parameters that can reflect bladder-related muscle function in urodynamic testing.

Primary outcomes were postvoided residual urine (PVR), maximal cystometric capacity (MCC), maximal flow rate (MFR) and bladder capacity for first voiding desire (BFD). The secondary outcomes included bladder function recovery rate (BR) and urinary tract infection rate (UIR). Safety outcomes are reports of related adverse events (AE).
