# Supplementary figures and images for: Efficacy and safety of acupuncture for urinary retention after hysterectomy: A systematic review and meta-analysis
Source: Medicine (Baltimore). 2021 Jun 4;100(22):e26064. doi: 10.1097/MD.0000000000026064 (PMC8183752; doi:10.1097/MD.0000000000026064)

**Supplementary Figure S1.** Risk of bias for the 12 RCTs in the meta-analysis


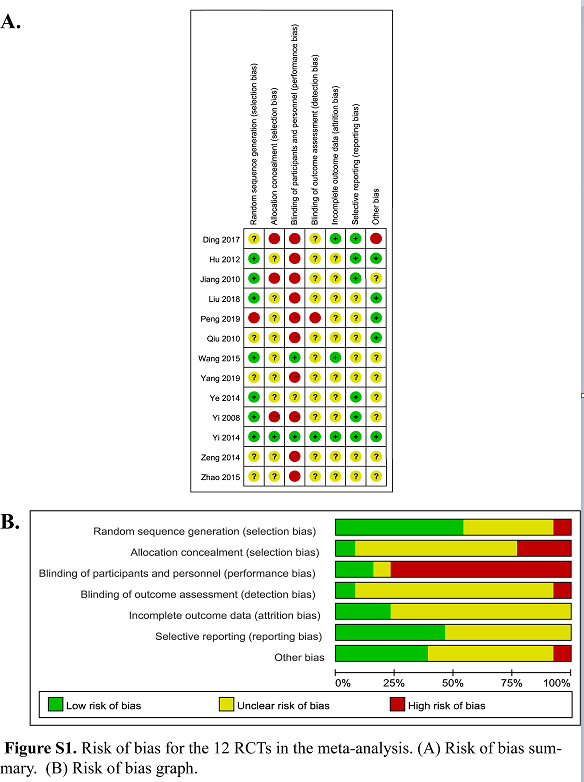

Supplement: Supplemental Digital Content [file medi-100-e26064-s003.doc]
