# Supplementary material for: Efficacy and safety of acupuncture for urinary retention after hysterectomy: A systematic review and meta-analysis
Source: Medicine (Baltimore). 2021 Jun 4;100(22):e26064. doi: 10.1097/MD.0000000000026064 (PMC8183752; doi:10.1097/MD.0000000000026064)
Supplement: Supplemental Digital Content [file medi-100-e26064-s004.doc]

| **Supplementary Table S1. Summary of findings in GRADE** |
| --- |

| **Acupuncture compared to control for UR after hysterectomy woman** | | | | | | |
| --- | --- | --- | --- | --- | --- | --- |
| **Patient or population**: UR after hysterectomy  **Setting**:  **Intervention**: acupuncture  **Comparison**: control | | | | | | |
| Outcomes | **Anticipated absolute effects^*^** (95% CI) | | Relative effect (95% CI) | № of participants  (studies) | Certainty of the evidence (GRADE) | Comments |
|  | **Risk with control** | **Risk with acupuncture** |  |  |  |  |
| PVR | - | MD **25.59** **lower** (30.45 lower to 20.73 lower) |  | 975 (11 RCTs) | ⨁⨁◯◯ **Low** ^a,d^ |  |
| MCC |  | MD **39.54** (10.30 to 68.78) |  | 380 (4 RCTs) | ⨁⨁◯◯ **Low** ^d,g^ |  |
| MFR | - | MD **7.58** (5.19 to 9.97) | - | 516 (5 RCTs) | ⨁◯◯◯ **Very Low** ^d,e,g^ |  |
| BFD | - | MD **61.98 lower** (90.69 lower to 33.26 lower) | - | 392 (3 RCTs) | ⨁⨁◯◯ **Low** ^d,e^ |  |
| BR | 656 per 1000 | **916 per 1000**  **(774 to 1429)** | **RR 1.36** (1.18 to 1.56) | 749 (9 RCTs) | ⨁◯◯◯ **Very Low** ^a,f,g^ |  |
| UIR | 162 per 1,000 | **29 per 1,000** (45 to 183) | **RR 0.22** (0.06 to 0.82) | 136 (2 RCTs) | ⨁⨁◯◯ **Low** ^b,c,h^ |  |
| ***The risk in the intervention group** (and its 95% confidence interval) is based on the assumed risk in the comparison group and the **relative effect** of the intervention (and its 95% CI).   **CI:** Confidence interval; **RR:** Risk ratio; **OR:** Odds ratio; **MD:** Mean difference; **SMD:** Standardised mean difference | | | | | | |
| **GRADE Working Group grades of evidence** **High certainty:** We are very confident that the true effect lies close to that of the estimate of the effect **Moderate certainty:** We are moderately confident in the effect estimate: The true effect is likely to be close to the estimate of the effect, but there is a possibility that it is substantially different **Low certainty:** Our confidence in the effect estimate is limited: The true effect may be substantially different from the estimate of the effect **Very low certainty:** We have very little confidence in the effect estimate: The true effect is likely to be substantially different from the estimate of effect | | | | | | |

#### Explanations

a. One study has a small sample size.

b. Few included studies are only two.

c. After the merger, the heterogeneity is small.

d. After the merger, the overall heterogeneity is large.

e. One study is published as doctorate thesis.

f. After the merger, the heterogeneity is moderate.

g. After the merger, sensitivity analysis found that the risk of bias was greater.

h. The relevant decision-making criteria are ambiguous.
