# Supplementary material for: Efficacy and safety of acupuncture for urinary retention after hysterectomy: A systematic review and meta-analysis
Source: Medicine (Baltimore). 2021 Jun 4;100(22):e26064. doi: 10.1097/MD.0000000000026064 (PMC8183752; doi:10.1097/MD.0000000000026064)
Supplement: Supplemental Digital Content [file medi-100-e26064-s005.doc]

**Supplementary Table S2. Sensitivity analysis of each outcome.**

| **Outcome** | **All studies** |  |  | **Sensitivity analysis^1^** | | | **Results** |
| --- | --- | --- | --- | --- | --- | --- | --- |
|  | **N. Studies/**  **participant** | **RR (95%CI) or**  **MD (95%CI) or SMD (95%CI)** | ***I^2^*** | **N. Studies/**  **participant** | **RR (95%CI) or**  **MD (95%CI)** | ***I^2^*** |  |
| PVR | 11 / 975 | MD= -25.59 (-30.45, -20.73) | 91% | 10 / 847 | MD = -21.33 (-25.46, -17.2) | 88% | Not affected |
| MCC | 4 / 380 | MD = 39.54 (10.30, 68.78) | 90% | 3 / 260 | MD= 50.57 (27.71, 27.90) | 60% | affected△ |
| MFR | 5 / 516 | MD= 7.58 (5.19, 9.97) | 89% | 4 / 396 | MD= 8.25 (6.43, 10.06) | 67% | affected△ |
| BFD | 3 / 392 | MD = -61.98 (-90.69, -33.26) | 97% | 2 / 270 | MD = -75.36 (-128.24, -22.48) | 98% | Not affected |
| BR | 9 / 749 | RR = 1.36 (1.18, 1.56) | 69% | 8 / 873 | MD= 1.28 (1.16, 1.41) | 40% | affected† |
| UIR | 2 / 136 | RR = 0.22 (0.06, 0.82) | 0% | N/A | N/A | N/A | N/A |

^1^ Sensitivity analysis excluding studies judged to be at high risk of bias evaluated by funnel plot asymmetry

RR: relative risk

MD: mean difference

SMD: standardized mean difference

△The source of heterogeneity is mainly related to the long interval between intervention and check points in Yi 2014 (19 days between the end of the intervention and the monitoring point).

†The source of its heterogeneity is mainly related to the late acupuncture intervention in Zhao 2015 (within 5 days after surgery vs. 14 days after surgery).
